# The Role of Donor Milk and Human Milk Banks in Promoting Maternal and Infant Health: Positive Contributions from the Articles of the Special Issue

**DOI:** 10.3390/nu18152516

**Published:** 2026-08-03

**Authors:** Guido E. Moro, Enrico Bertino, Alessandra Coscia

**Affiliations:** 1 Italian Association of Human Milk Banks (AIBLUD = Associazione Italiana Banche del Latte Umano Donato), 20126 Milan, Italy; enrico.bertino@unito.it; 2 Neonatal Intensive Care Unit, Department of Public Health and Pediatrics, University of Turin, 10126 Turin, Italy; alessandra.coscia@unito.it

## 1. Introduction

The special issue *The Role of Donor Milk and Human Milk Banks in Promoting Maternal and Infant Health* offers a comprehensive overview of the growing scientific evidence supporting human milk donation and the development of human milk banks. The first Human Milk Bank (HMB) opened in Wien in 1909, and today more than 700 HMBs are active all over the world. Many differences exist among HMBs in management, operational procedures, quality controls and other practical aspects, and all these differences may influence the efficacy and efficiency of HMBs [1]. In spite of these differences, HMBs play a fundamental role in promoting maternal and infant health [2]. The eight articles included in the issue address complementary aspects of neonatal nutrition, lactation support, human milk processing, growth, neurodevelopment, and organizational models of care.

Taken together, these studies demonstrate that donor human milk and human milk banks extend far beyond the simple provision of nutrition. They constitute an integrated system capable of improving neonatal outcomes, supporting breastfeeding, promoting maternal health, and strengthening family-centered care, particularly for preterm and vulnerable infants.

## 2. Comments on the Articles

Contribution 1, *Long-Term Neurodevelopmental Outcomes of Late Preterm Children: A Pilot Study on the Role of Early Nutrition* by Augusto Biasini et al., highlights the importance of nutritional strategies during the neonatal period. The study suggests that the quality of early nutrition may influence later neurodevelopmental outcomes in late preterm infants.

The positive message emerging from this work is that human milk exposure during the critical early period may contribute to improved cognitive and developmental trajectories. The study reinforces the concept that nutrition acts as a form of developmental care, influencing brain maturation during a particularly vulnerable stage of life.

By emphasizing long-term outcomes rather than short-term growth alone, the study broadens the perspective of neonatal nutrition and supports the use of human milk as an investment in future health and neurodevelopment.

Contribution 2, *Donor Human Milk, Formula Supplementation, and Bone Metabolism*, by Jacky Herzlich et al., evaluating donor human milk and formula supplementation in preterm infants receiving their mother’s own milk, addresses an important clinical question: how different supplementary feeding strategies influence bone metabolism and neonatal outcomes.

Its principal contribution is the demonstration that donor milk can be safely integrated into feeding protocols without compromising clinical outcomes. The study supports donor milk as the preferred supplement when maternal milk is insufficient.

This finding is particularly important because it strengthens confidence among clinicians that donor milk can replace formula during periods of limited maternal supply while maintaining satisfactory nutritional and metabolic results. The article therefore reinforces international recommendations that donor milk should be the first alternative to maternal milk.

Contribution 3, *The Fourth National Survey* on Italian human milk banks, from Giuseppe De Nisi et al., provides valuable epidemiological and organizational information. The article documents the growth and consolidation of milk banking activities throughout Italy.

The survey demonstrates that milk banking has become an established component of neonatal care in Italy. The existence of a coordinated network improves access to donor milk and reduces inequalities in care.

Furthermore, the survey provides health authorities with essential data for planning future services, supporting the expansion of milk banks and encouraging national policies that promote breastfeeding and donor milk use.

Contribution 4, *Bridging the Milk Gap*, by Jacqueline Barin et al., proposes an innovative organizational model by comparing human milk banking with blood banking systems. A partnership between blood collection centers and HMBS, particularly for specific items like screening protocols and storage of milk products, combined with clear regulatory aspects, could be utilized universally in order to improve the efficiency of the collection system for HMBs [3]. Besides, the recent inclusion of donor human milk in the list of substances of human origin (SoHO), may bring additional safety and improve the quality of the final product of HMBs [4].

The positive contribution of this work lies in its recognition of donor human milk as a valuable biological resource requiring professional management, quality assurance, traceability, and equitable distribution.

The blood-bank model offers several advantages: standardized procedures; improved logistics; enhanced safety controls; better donor management; and stronger institutional support.

This approach elevates human milk banking from a voluntary activity to an essential healthcare service. The article also highlights the importance of integrating milk banks into broader healthcare systems, thereby increasing sustainability and accessibility.

Ultimately, the paper promotes a vision in which donor milk becomes a recognized component of neonatal medicine, supported by robust organizational structures.

One of the most encouraging findings in this special issue comes from Contribution 5, *Three Years of Human Milk Banking and Breastfeeding Rates at Discharge*, by Federica Mongelli et al., evaluating three years of milk banking activity in a tertiary Italian neonatal intensive care unit.

The investigators observed that the presence of a milk bank was associated with improved lactation outcomes and higher breastfeeding rates at discharge among very low birth weight infants.

This result is particularly significant because some professionals have historically feared that donor milk availability might reduce maternal motivation to breastfeed. The study demonstrates the opposite.

Human milk banks appear to support maternal lactation, reduce parental stress, allow mothers additional time to establish milk production, and encourage breastfeeding continuation.

The milk bank therefore functions not only as a nutritional resource but also as a powerful lactation support strategy. The article confirms that donor milk programs and breastfeeding promotion are mutually reinforcing rather than competing interventions.

Contribution 6, *Enteral Nutrition and Clinical Outcomes in Very Low Birth Weight Infants,* by Pasqua Anna Quitadamo et al., is a retrospective cohort study examining enteral nutrition and clinical outcomes in very low birth weight infants, which provides additional evidence regarding the importance of early nutritional strategies.

The study demonstrates that optimized enteral nutrition is associated with improved clinical outcomes and better tolerance of feeding advancement.

Human milk, either maternal or donor, plays a central role in the nutritional protocols of these tiny infants. The study reinforces the concept that neonatal nutrition should be considered a therapeutic intervention capable of influencing morbidity and recovery.

This work contributes to the special issue by emphasizing the clinical importance of feeding practices and supporting protocols that prioritize human milk.

Contribution 7, on *High-Temperature Short-Time Pasteurization versus Conventional Methods*, by Nadia Raquel Garcia-Lara et al., shows that pasteurization is essential for the safety of donor human milk, but the process may affect certain biological properties.

The study is comparing high-temperature short-time pasteurization with traditional methods in extremely low birth weight infants and provides important evidence that technological innovations can improve milk quality.

The findings suggest that advanced processing techniques may optimize the balance between microbiological safety and biological activity.

This article demonstrates that milk banks continue to evolve scientifically and technologically. By improving processing methods, milk banks may further enhance the benefits of donor milk for the most fragile infants.

The final article, Contribution 8, *Individualized Fortification of Human Milk*, by Manuela Cardoso et al., addresses individualized fortification based on measured macronutrient content of human milk.

This study represents an important step toward precision nutrition in neonatal care. Rather than assuming that all human milk contains identical nutrient concentrations, individualized analysis allows fortification to meet the specific needs of each infant.

The study reports improvements in growth; body composition; and nutritional adequacy.

These findings demonstrate that donor and maternal milk can be optimized to support the growth requirements of very preterm infants while preserving the biological advantages of human milk.

Individualized fortification reconciles two major goals of neonatal nutrition: promoting growth and maintaining the protective properties of human milk.

## 3. Overall Contributions of the Special Issue

Although the eight studies investigate different topics, several common themes emerge.

1. **Human milk is more than nutrition.** Many studies emphasize the biological, developmental, immunological, and neurodevelopmental effects of human milk.
2. **Donor milk is a safe and effective alternative.** When maternal milk is unavailable or insufficient, donor milk provides clinically important benefits and may reduce dependence on formula.
3. **Human milk banks support breastfeeding.** Rather than replacing maternal milk, milk banks facilitate lactation and improve breastfeeding outcomes.
4. **Organization matters.** National networks, standardized procedures, and integration into healthcare systems increase the effectiveness and sustainability of milk banking programs.
5. **Innovation continues.** Advances in pasteurization techniques, nutritional analysis, and individualized fortification are improving the quality of donor milk services.

## 4. Conclusions

The eight articles included in this special issue collectively demonstrate that donor human milk and human milk banks have become essential components of modern neonatal care. Their impact extends beyond the prevention of disease and includes improvements in neurodevelopment, growth, breastfeeding success, nutritional management, and healthcare organization.

The studies provide strong evidence that human milk banks promote both maternal and infant health. They support mothers during periods of lactation difficulty, ensure equitable access to human milk for vulnerable infants, and contribute to better short-term and long-term outcomes. As a consequence, several efforts should be done to improve the knowledge of mothers regarding donor human milk and milk banks that is still very limited [5].

There is a vast amount of evidence that shows the role of donor human milk banking in supporting internationally the human rights of the world’s most vulnerable infants. These documents demonstrate that the use of banked human donor milk represents a human right issue, able to improve the health of human beings during childhood and adult life [6,7].

Overall, the special issue portrays donor milk banking as an integrated model of care that combines scientific evidence, technological innovation, clinical excellence, and family support. Its findings strongly support continued investment in human milk banks and the expansion of donor milk programs worldwide.
